# Subcutaneous preconditioning increases invasion and metastatic dissemination in mouse colorectal cancer models

**DOI:** 10.1242/dmm.013995

**Published:** 2014-01-30

**Authors:** Patricia Alamo, Alberto Gallardo, Miguel A. Pavón, Isolda Casanova, Manuel Trias, Maria A. Mangues, Esther Vázquez, Antonio Villaverde, Ramon Mangues, Maria V. Céspedes

**Affiliations:** 1 Oncogenesis and Antitumour Drug Group, Biomedical Research Institute Sant Pau (IIB-SantPau), Hospital de la Santa Creu i Sant Pau, C/Sant Antoni Maria Claret, 167, 08025 Barcelona, Spain.; 2 CIBER de Bioingeniería, Biomateriales y Nanomedicina (CIBER-BBN), 08025 Barcelona, Spain.; 3 Department of Pathology, Clínica Girona, C/Joan Maragall, 26, 17002 Girona, Spain.; 4 Department of General and Digestive Surgery, Hospital de la Santa Creu i Sant Pau, 08025 Barcelona, Spain.; 5 Department of Pharmacy, Hospital de la Santa Creu i Sant Pau, 08025 Barcelona, Spain.; 6 Institut de Biotecnologia i de Biomedicina, Universitat Autònoma de Barcelona and Department de Genètica i de Microbiologia, Universitat Autònoma de Barcelona, Bellaterra, 08193 Barcelona, Spain.

**Keywords:** Collective invasion, Colorectal cancer model, Metastasis, Orthotopic injection, Single tumour cell, Subcutaneous preconditioning

## Abstract

Mouse colorectal cancer (CRC) models generated by orthotopic microinjection of human CRC cell lines reproduce the pattern of lymphatic, haematological and transcoelomic spread but generate low metastatic efficiency. Our aim was to develop a new strategy that could increase the metastatic efficiency of these models. We used subcutaneous implantation of the human CRC cell lines HCT116 or SW48 prior to their orthotopic microinjection in the cecum of nude mice (SC+ORT). This subcutaneous preconditioning significantly enhanced metastatic dissemination. In the HCT116 model it increased the number and size of metastatic foci in lymph nodes, lung, liver and peritoneum, whereas, in the SW48 model, it induced a shift from non-metastatic to metastatic. In both models the number of apoptotic bodies in the primary tumour in the SC+ORT group was significantly reduced compared with that in the direct orthotopic injection (ORT) group. Moreover, in HCT116 tumours the number of keratin-positive tumour buddings and single epithelial cells increased at the invasion front in SC+ORT mice. In the SW48 tumour model, we observed a trend towards a higher number of tumour buds and single cells in the SC+ORT group but this did not reach statistical significance. At a molecular level, the enhanced metastatic efficiency observed in the HCT116 SC+ORT model was associated with an increase in AKT activation, VEGF-A overexpression and downregulation of β1 integrin in primary tumour tissue, whereas, in SW48 SC+ORT mice, the level of expression of these proteins remained unchanged. In summary, subcutaneous preconditioning increased the metastatic dissemination of both orthotopic CRC models by increasing tumour cell survival and invasion at the tumour invasion front. This approach could be useful to simultaneously study the mechanisms of metastases and to evaluate anti-metastatic drugs against CRC.

## INTRODUCTION

Most established human carcinoma cell lines that are used to develop cancer models are adapted to *in vitro* conditions and present a low metastatic rate after their orthotopic microinjection in immunosuppressed mice. When metastases are achieved, only microfoci are observed, limiting the analysis and clinical translation of the findings (Céspedes et al., 2006). Previous reports have described the role of the microenvironment in regulating tumour progression and metastatic dissemination in animal models (Joyce and Pollard, 2009; McAllister and Weinberg, 2010). Bruns and coworkers showed that successive re-injection of cells from hepatic metastases of pancreatic cancer biopsies into the pancreas or spleen of nude mice increases the yield of lymphatic and hepatic metastases compared with injection of parental cells (Bruns et al., 1999). Other authors have found that serial *in vivo* passage of tumours creates more-aggressive variants of human cancer cells in animal models, but these can take around a year to develop (Bruns et al., 1999; Shah et al., 2006).

We previously developed a metastatic cancer model by orthotopic microinjection of human colorectal cancer (CRC) cells that disseminate to all clinically relevant sites (lymph nodes, liver, lung and peritoneum) (Céspedes et al., 2007). Most of the metastatic foci generated in this model were microscopic, and only a few macrometastases or visible metastases were observed, confined to the mesenteric lymph nodes and peritoneal cavity.

Our aim was to improve this orthotopic model by performing a prior single subcutaneous passage of HCT116 or SW48 CRC cells. We hypothesized that subcutaneous (SC) preconditioning before orthotopic microinjection would increase their metastatic efficiency. We also expected that the generated models would be useful to study the mechanisms of metastases and the preclinical development of novel anti-metastatic drugs.

## RESULTS

### Subcutaneous preconditioning enhanced the metastases, without altering colonic tumour growth

There were no differences in the percentage of tumour engraftment between groups that underwent implantation of the human CRC cell lines HCT116 or SW48 (SC preconditioning) prior to their orthotopic microinjection (ORT) in the cecum of nude mice (SC+ORT) and ORT groups in the HCT116 (78% vs 100%, respectively) or SW48 (33% vs 14%, respectively) models. Moreover, we did not observe significant differences in primary tumour volume at the end of the experiment between the SC+ORT (1158±215 mm^3^) and ORT (1275±299 mm^3^) groups for HCT116, or between the SC+ORT (940.5±57.5 mm^3^) and ORT (940 mm^3^) groups for SW48. The SC+ORT and ORT groups in HCT116 and SW48 mice both developed undifferentiated tumours, with 40–70% necrosis and a high degree of vascular invasion. No significant differences were observed in mouse survival between the SC+ORT and the ORT groups for the HCT116 (69±5 vs 67±8 days, respectively) or SW48 (187.3±35.7 vs 156 days, respectively) models.

**RESOURCE IMPACT**

**Background**

The majority of deaths associated with colorectal cancer (CRC) are attributable to metastatic disease. Many patients present with metastatic, late-stage CRC at the time of diagnosis, and this can prove difficult to treat. However, current mouse models of CRC, generated by orthotopic microinjection of human CRC cell lines, rarely reproduce advanced stages of the disease and generate low metastatic efficiency. Thus, their use for understanding the mechanisms underlying metastasis and testing the efficacy of potential anti-metastatic therapies is limited.

**Results**

In this study, the authors show that subcutaneous preconditioning prior to orthotopic injection of the HCT116 or SW48 CRC cell line in the cecum of nude (immunodeficient) mice generates models with enhanced metastatic efficiency in all clinically relevant sites: lymph nodes, liver, lung and peritoneum. Most importantly, macroscopic metastases in the liver, detected after injection of the HCT116 cell line, compromise liver function and lead to mouse death, recapitulating the progression of CRC in humans. These findings suggest that subcutaneous preconditioning induces a reduction in the rate of tumour cell death within primary tumours and an increased invasion at the tumour front, possibly explaining the increased dissemination and aggressiveness observed in the resulting models.

**Implications and future directions**

This study provides a novel procedure that increases the metastatic efficiency of current CRC mouse models and reproduces the metastatic dissemination observed in humans. Given that metastatic rate is enhanced in two independent CRC models using this procedure, it is likely that subcutaneous preconditioning could improve the metastatic efficiency in other cancer models. The *in vivo* models generated using this new approach could facilitate the study of the mechanisms of tumour invasion and metastatic dissemination and be used in the preclinical evaluation of anti-tumour and anti-metastatic compounds.

Whereas no differences were observed in primary tumour growth or mouse survival, the number of HCT116 mice bearing lung metastases was significantly (*P*<0.05) higher in the SC+ORT than in the ORT group. No significant differences between groups were recorded regarding the number of mice affected by liver or lymph node metastases, although the number and size of metastases were increased in these organs (see below). The direct orthotopic injection of the SW48 cell line (SW48 ORT) did not generate metastases in any of the injected animals. However, the SW48 SC+ORT group developed multiple metastases in the lymph nodes, lung and peritoneum. Thus, HCT116 SC+ORT mice had an enhanced metastatic rate and maintained the organo-tropism observed in the ORT group. Moreover, SC preconditioning switched the non-metastatic SW48 cell line to metastatic: SC+ORT SW48 mice developed metastases in most of the expected sites for CRC (lymph nodes, lung and peritoneum).

### SC preconditioning enhanced metastatic yield at all clinically relevant sites

**Fig. 1.**
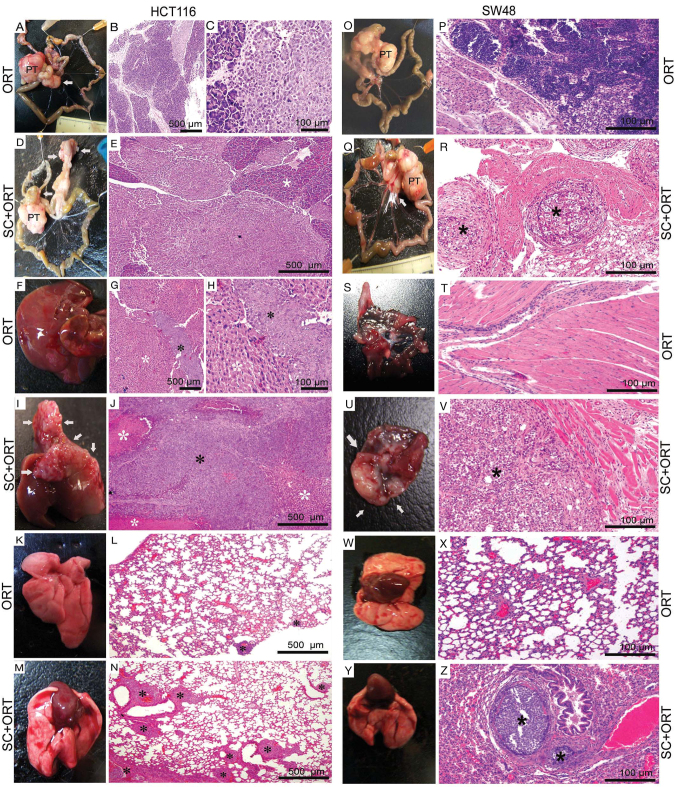
**Enhanced metastatic efficiency in SC+ORT mice.** (A–E, O–R) Lymph node metastases: the HCT116-ORT group showed micro- and macrometastasis (B, C), and visible lymph node metastases (A) that were smaller than those in the SC+ORT group. We recorded a low number of visible metastases (white arrow), involving mainly mesenteric lymph nodes (A), and only one visible peripancreatic lymph node. In contrast, in the HCT116-SC+ORT group (D,E), we observed a high number of visible metastases, affecting the peripancreatic parenchyma and the mesenteric lymph nodes (white arrows, D). In addition to the isolated visible metastases in the SC−ORT group, four animals developed a massive conglomerate of peripancreatic lymph node metastases (white arrows) involving the whole pancreas (D); these metastases were physically connected under the microscope (white asterisk, E). SW48 ORT mice lacked lymphatic dissemination (L,O). In contrast, SW48 SC+ORT mice showed lymphatic micro, macro and visible dissemination (white arrow in Q shows visible metastases and asterisks in R show micrometastases; Q,R). PT, primary tumour. (F–J) Liver metastases: in the liver of mice in the HCT116-ORT group (F–H), all metastases were microscopic (G,H, black asterisks). In contrast, in the HCT116-SC+ORT group we observed a larger number of microscopic metastases than in the ORT group, in addition to macroscopic and single visible metastases. Moreover, in two of the animals in the SC+ORT group (I,J), massive visible liver metastases developed (white arrows), forming a conglomerate (I) that invaded the hepatic parenchyma (white asterisks) and involved 95% of its area. Black asterisk in J shows macrometastasis. No liver metastases were observed in any group of SW48 mice (not shown). (S–V) Peritoneal metastases: no metastases were detected in the SW48-ORT group (S,T). In contrast, SW48 SC+ORT mice developed micro- and visible metastases (U,V, white arrows and black asterisk). (K–N,W–Z) Lung metastases: mice in the HCT116-SC+ORT group (M,N) developed a significantly higher number of microfoci in their lung (N, black asterisks) than mice in the ORT (K,L) group. In the SW48-ORT group, no pulmonary metastases were recorded (W,X). In contrast, SW48 SC+ORT mice developed micro- and macrometastases in the lung (Y,Z). Type of metastasis as a function of its diameter: microfoci <1 mm; macrofoci 1–3 mm; visible >3 mm; H&E staining. Scale bars for magnification.

HCT116 SC+ORT mice displayed enhanced efficiency of lymph node metastases, as compared with ORT mice (Fig. 1A–E; Table 1). We recorded a total of 83 visible lymph node metastases (19 mesenteric, 64 peripancreatic) in the SC+ORT mice (Fig. 1D), whereas, in the ORT group we only observed 26 (21 mesenteric, 5 peripancreatic) (Fig. 1A–C). This difference was statistically significant (*P*=0.003). HCT116 SC+ORT mice also developed significantly (*P*=0.020) larger visible metastases (790.7×10^5^±148.1×10^5^ μm^2^) than ORT mice (265.3×10^5^±38.8×10^5^ μm^2^) (Table 1). Moreover, in the SC+ORT group, four of the animals developed a massive conglomerate of peripancreatic lymph node metastases involving the whole pancreas (Fig. 1D,E) and invading most of the parenchyma. This was not observed in the ORT group, where metastatic foci were smaller (Fig. 1B,C; Table 1).

**Table 1.**
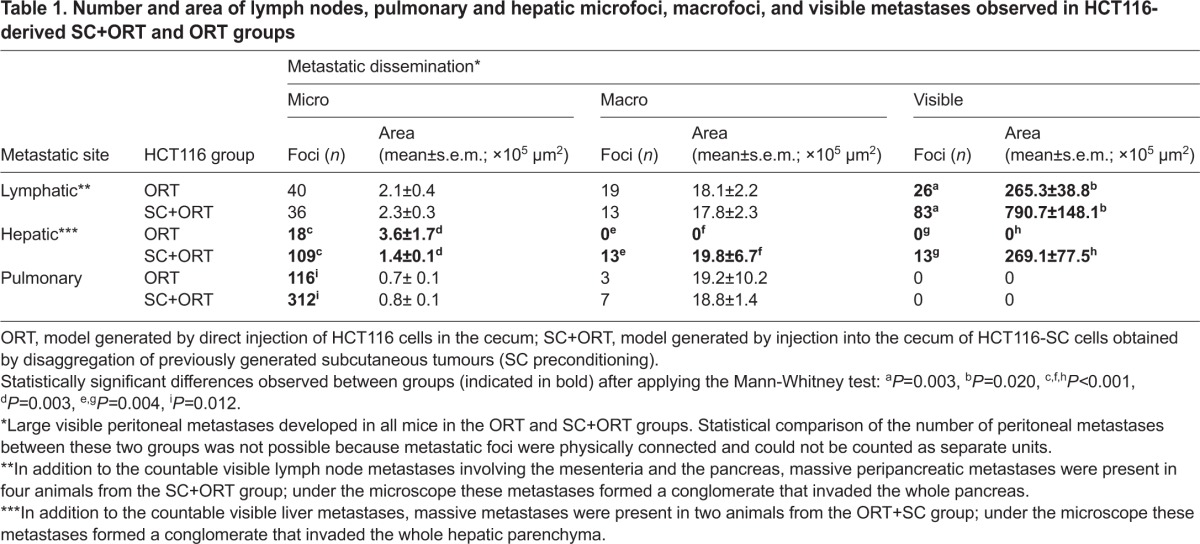
Number and area of lymph nodes, pulmonary and hepatic microfoci, macrofoci, and visible metastases observed in HCT116-derived SC+ORT and ORT groups

We also observed an enhanced efficiency of liver metastases in HCT116 SC+ORT mice as compared with the ORT group (Fig. 1F–J; Table 1). The number of liver metastases in the SC+ORT (*n*=135) group was significantly (*P*≤0.01) higher than in the ORT group (*n*=18), which showed only micro- and macro-metastases, but no visible metastases. In the SC+ORT group, we identified 13 single visible metastases with an average size of 269.1×10^5^±77.5×10^5^ μm^2^ (Table 1). Massive visible liver metastases developed in two mice in the SC+ORT group; these metastases formed a conglomerate (Fig. 1I,J) that invaded the hepatic parenchyma, involving 95% of its area according to microscopic analysis. We also observed 13 macroscopic metastases of an average size of 19.8×10^5^±6.7×10^5^ μm^2^ in the SC+ORT group. The number of microfoci in this group (*n*=109) was significantly (*P*<0.01) higher than in the ORT group (*n*=18). Although mouse survival did not differ between groups, the two mice that developed massive liver metastases in the SC+ORT group had a shorter survival time (59±5 days) than the remaining animals (69±5 days), suggesting that liver metastases, rather than primary tumour growth, induced death in these mice.

Lung dissemination in HCT116 tumour-bearing mice was also enhanced in SC+ORT mice as compared with ORT mice (Fig. 1KN; Table 1). The SC+ORT group developed a total of 312 microfoci, a number significantly (*P*=0.012) higher than the 116 recorded in the ORT group. Moreover, the percentage of mice affected by lung metastases was also significantly (*P*<0.05) higher in the SC+ORT (89%) than in the ORT (30%) group. No other differences in lung metastases were recorded; thus, the number and size of macrofoci did not differ significantly between groups. No visible lung metastases were detected in either group (Fig. 1K–N; Table 1). Visible and large peritoneal metastases were seen in ORT and SC+ORT mice. Nevertheless, we were unable to compare the efficiency of peritoneal dissemination between groups because, under the microscope, metastases were seen as physically connected and could not be counted as separate units.

As stated above, SW48 ORT mice did not develop metastases. In contrast, the SC preconditioning of SW48 cells induced the development of multiple metastases at the lymph nodes, lung and peritoneum in the SC+ORT group, a highly significant difference for each metastatic site (*P*<0.05, Table 2). Thus, in the SC+ORT group we recorded 19 lymph node metastases (8 micro-, 7 macro- and 4 of a visible size), their sizes averaging 1.1×10^5^±0.6×10^5^, 20.1×10^5^±3.1×10^5^ and 512.7×10^5^±309×10^5^ μm^2^, respectively (Fig. 1O–R; Table 2). The total number of peritoneal metastases in the SC+ORT group (Fig. 1S–V; Table 2) was 17 (2 micro- and 15 visible metastases), their size being 0.2×10^5^±0.02×10^5^ and 388.9×10^5^±116.3×10^5^ μm^2^ (Table 2). Finally, the total number of foci recorded in the lung in the SC+ORT group was 28 (26 micro-, 2 macro- and no visible metastases), their size averaging 0.8×10^5^±0.003×10^5^ and 18.5×10^5^±0.09×10^5^ μm^2^, respectively (Fig. 1W–Z; Table 2).

**Table 2.**
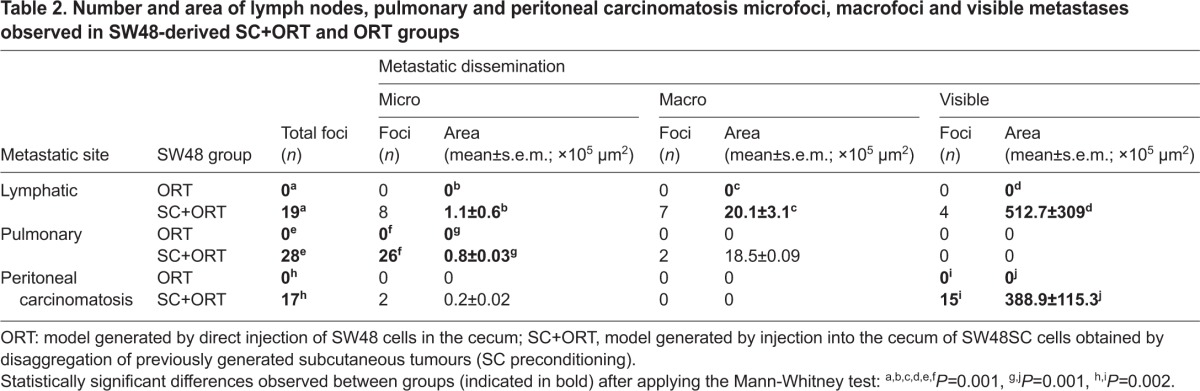
Number and area of lymph nodes, pulmonary and peritoneal carcinomatosis microfoci, macrofoci and visible metastases observed in SW48-derived SC+ORT and ORT groups

All primary tumours and metastatic foci from SW48 and HCT116 mice were verified to be human-origin tumour cells (HCT116 or SW48) by using the human-specific antibody MHC-1 class I (supplementary material Fig. S1).

### SC preconditioning decreased cell death and increased invasion in primary tumours

**Fig. 2.**
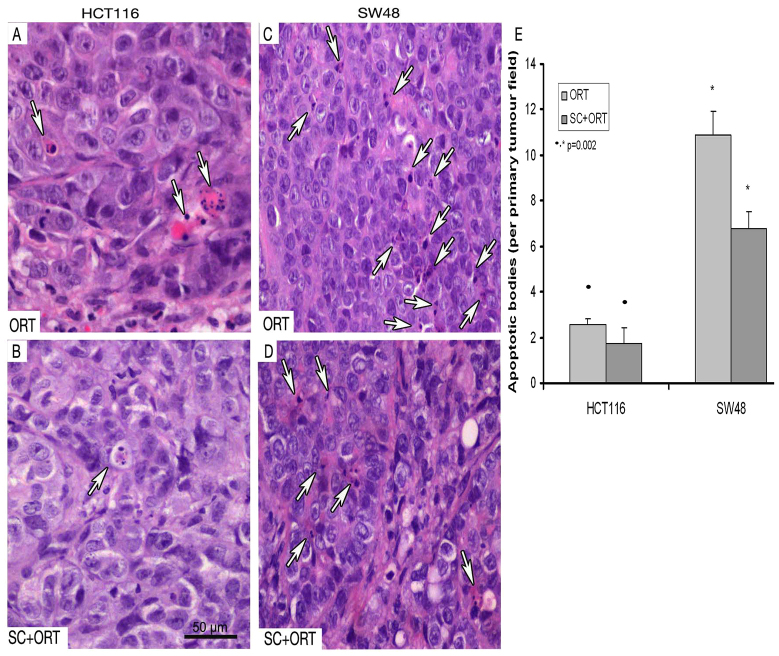
**Increased number of apoptotic bodies in the primary tumour in the ORT groups.** The primary tumours in the HCT116-ORT group (A) showed a significantly (*P*=0.002) higher number of apoptotic cells (white arrows) than primary tumours in the HCT116-SC+ORT group (B,E). Primary tumours in the SW48-ORT group (C) also displayed an increased number of apoptotic bodies (white arrows) as compared with the SW48-ORT group (D,E). Quantification of apoptotic bodies was performed in a 400× magnified primary tumour field. Scale bar for magnification (A–D all have the same magnification).

The rate of cell death assessed in histology sections of primary tumours that developed in HCT116 SC+ORT mice was lower than in ORT mice. Thus, the SC+ORT group displayed a significantly (*P*=0.002) lower (1.74±0.25) number of apoptotic figures than the ORT (2.58±0.25) (Fig. 2A,B,E). Similarly, the number of apoptotic figures in the SW48 SC+ORT group (6.8±0.7) was significantly lower (*P*=0.002) than in the ORT group (10.9±1) (Fig. 2C–E). Interestingly, the apoptotic rate in both ORT and SC+ORT groups in SW48 mice was higher than in HCT116 mice (Fig. 2E; Table 3).

**Table 3.**
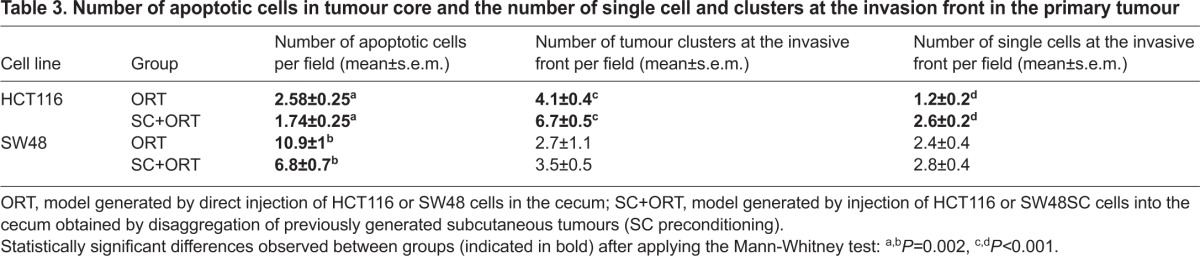
Number of apoptotic cells in tumour core and the number of single cell and clusters at the invasion front in the primary tumour

**Fig. 3.**
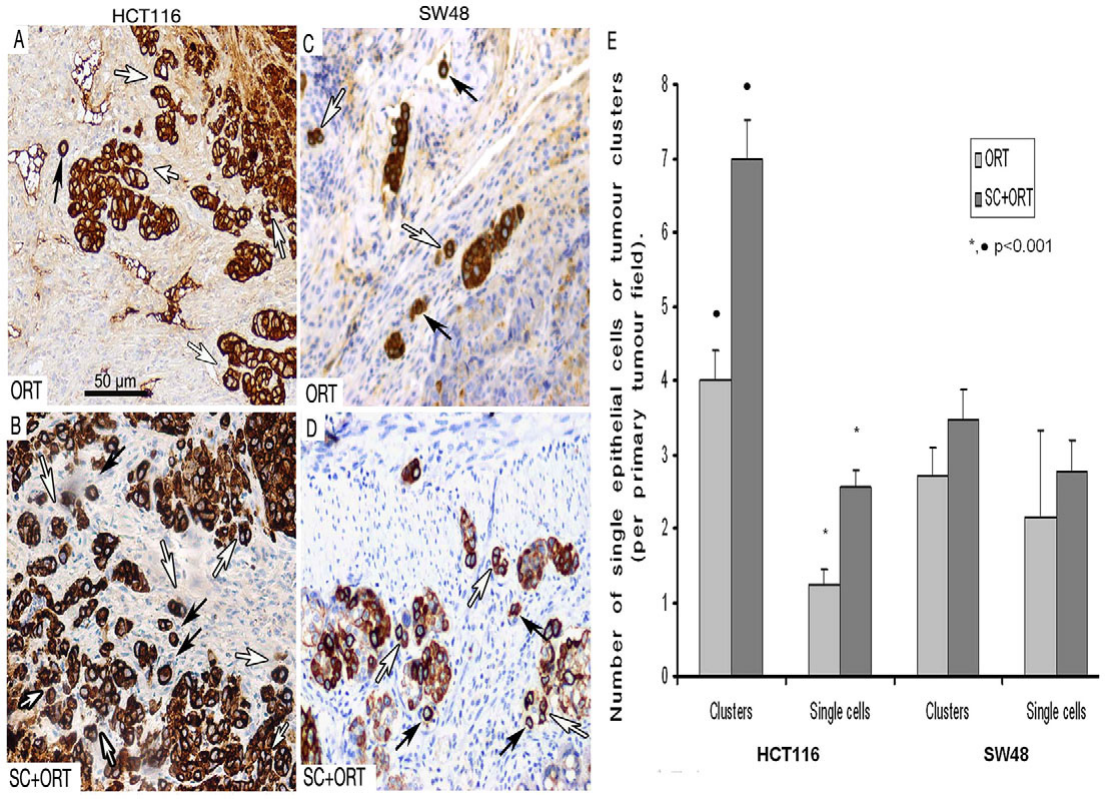
**Increased number of tumour cell clusters and single cells at the primary tumour invasive front in SC+ORT tumours.** The primary tumours in the HCT116-SC+ORT group (B) showed a significantly (*P*<0.001) larger number of pan-keratin-positive small clusters of tumour epithelial cells, surrounded by stroma (white arrows), than primary tumours in the HCT116-ORT group (A,E). Moreover, the number of pan-keratin-positive single tumour epithelial cells, completely surrounded by stroma, at the invasive front in HCT116-SC+ORT tumours (B, black arrows) was also significantly (*P*<0.001) higher than in ORT tumours (A,E). The tumour front in SW48-SC+ORT mice (D) displayed an increased number of clusters (white arrows) and single cells (black arrows) compared with the tumour front in SW48-ORT mice (C,E). (E) Quantification of tumour cell clusters of five or fewer cells (tumour clusters or budding) or single epithelial cells per 200× magnified primary tumour field observed in ORT and SC+ORT tumours. Scale bar for magnification (A–D all have the same magnification).

We also analyzed tumour budding and the number of single tumour epithelial cells at the primary tumour invasion front as a measure of the invasive capacity of the tumours. Thus, the number of tumour clusters at the invasion front (Fig. 3, white arrows) in the primary tumours of the HCT116 SC+ORT group (Fig. 3B) was significantly (*P*<0.001) higher than the number of clusters (6.7±0.5/field) in the ORT group (4.1±0.4/field) (Fig. 3A). Similarly, the number of single epithelial cells (Fig. 3, black arrows) at the invasion front in SC+ORT tumours (2.6±0.2/field) was significantly (*P*<0.001) higher than in ORT tumours (1.2±0.2/field) (Fig. 3E). Primary tumours in the SW48 SC+ORT group showed a higher number of clusters (3.5±0.5/field) at the tumour front than the ORT group (2.7±1.1/field) (Fig. 3C–E), but this difference was not significant (*P*=0.515). The number of single epithelial cells at the invasion front in SW48 SC+ORT tumours was also higher (2.8±0.4/field) than in the ORT tumours (2.4±0.4/field) (Fig. 3C–E), but, again, this trend did not reach statistical significance (*P*=0.137).

### SC preconditioning altered the expression of proteins that regulate survival and invasion in primary tumours

**Fig. 4.**
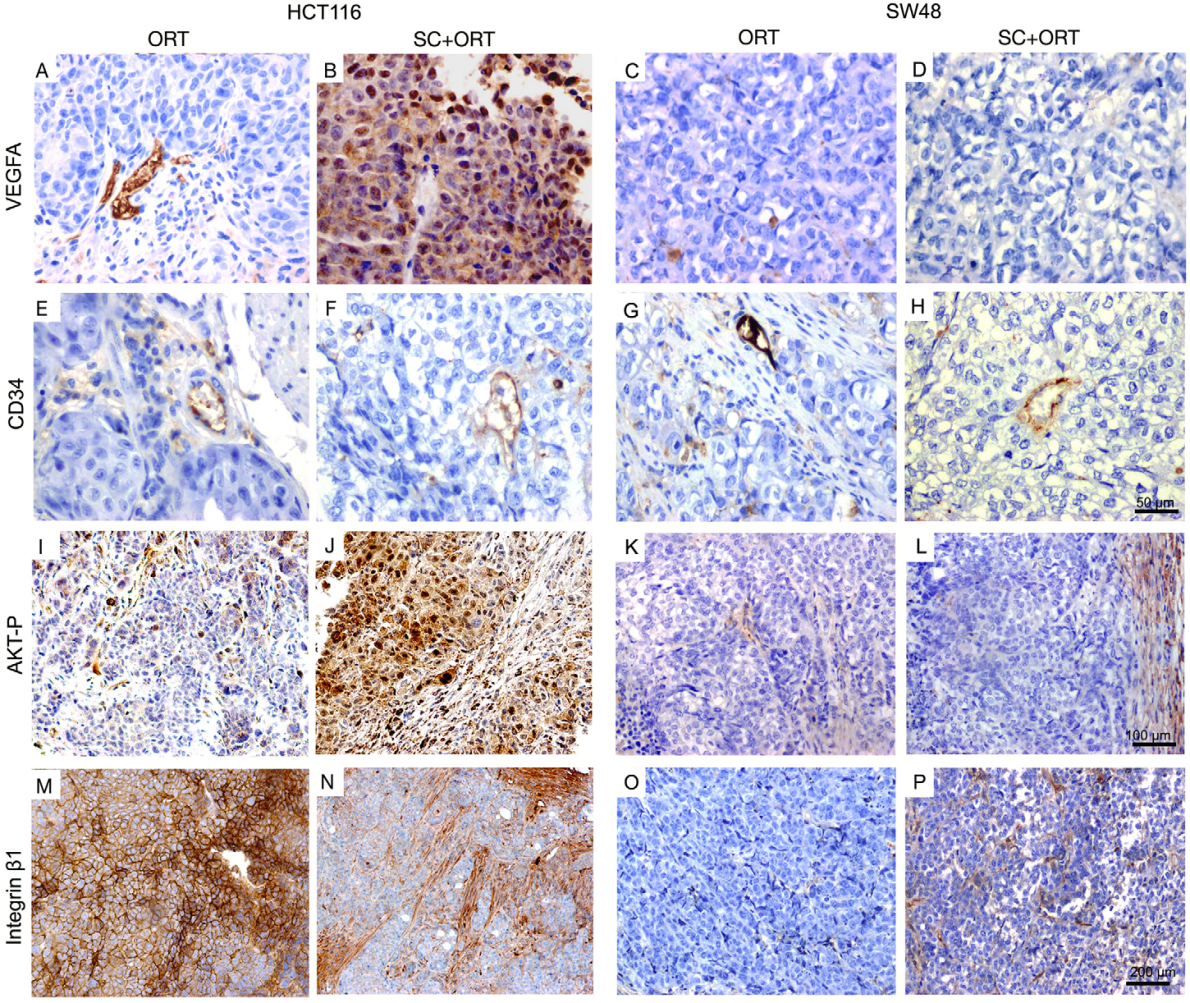
**Expression of proteins that regulate survival and invasion in primary tumours in ORT and SC+ORT groups.** No differences in the pattern and number of vessels, assessed by CD34 immunostaining, were observed between ORT and SC+ORT groups in HCT116 or SW48 mice (E–H). Interestingly, primary tumours in the HCT116-ORT (A) group showed significantly lower VEGFA expression than the SC+ORT group (B), whereas no VEGFA expression was observed in any of the SW48 groups (C,D). Primary tumours in the HCT116-ORT group (I) presented a lower level of AKT activation than that observed in HCT116 SC+ORT tumours (J). No differences in AKT activation were observed in primary tumours between ORT and SC+ORT groups in SW48 mice (K,L). HCT116-ORT primary tumours (M) showed a significantly higher level of β1 integrin expression than SC+ORT primary tumours (N), whereas no expression of β1 integrin was detected in primary tumours in any groups of SW48 mice (O,P). Scale bars for magnification (A–H, I–L and M–P have the same magnification).

Next, we evaluated whether the SC passage prior to the orthotopic injection changed the pattern of expression of several proteins that regulate cell survival, adhesion and invasion. VEGF-A expression in HCT116 SC+ORT primary tumours, as assessed by immunohistochemistry (IHC), was increased as compared with ORT tumours (Fig. 4A,B; supplementary material Table S1). We confirmed this finding by ELISA. Thus, primary tumour tissue in the SC+ORT group displayed a significantly (*P*=0.004) higher level of expression of VEGF-A than in the ORT group. In contrast, VEGF-A expression was absent in both the SC−ORT and ORT groups in SW48 primary tumours (Fig. 4C,D). The level of VEGF-A expression did not correlate with vascularization in tumours: the analysis of tumours using the blood-vessel marker CD34 showed a similar vascular pattern in SC+ORT and ORT groups in primary tumours of both HCT116 (Fig. 4E,F) and SW48 (Fig. 4G,H) xenografts.

Tumour cells in the primary site of the HCT116 SC+ORT group displayed a significantly higher level of activation of the AKT pathway (Fig. 4J; supplementary material Table S2) than in the ORT group (Fig. 4I; supplementary material Table S2). This activation was almost undetectable using IHC with an anti-p-AKT antibody in the SC+ORT and ORT groups in SW48-derived mice (Fig. 4K,L). The expression of the cell–cell-contact molecule E-cadherin did not differ significantly between groups within the bulk of the primary tumour or at their invasive front in HCT116 or SW48 mice. Out of the 13 evaluated integrins (α:1,2,3,4,5,6 and ν and β:1,2,3,4,5 and 6), only β1 showed a differential expression. HCT116 primary tumours in the SC+ORT group expressed significantly (*P*<0.01) lower levels of β1 integrin than in the ORT group (Fig. 4M,N; supplementary material Table S2), whereas, in SW48 tumours, no differences between groups were found (Fig. 4O,P). Expression of this protein in HCT116 tumours was thus downregulated by the SC passage, because both the SC tumours that generated the HCT116-SC cells and the SC+ORT primary tumours derived from their orthotopic injection showed low levels of β1 integrin expression. This finding was in contrast to the high level of expression of this protein in the HCT116 cultured cell line and in ORT primary tumours derived from their orthotopic injection (data not shown).

The level of expression of the metalloproteinase MMT1-MMP was not significantly different between groups (data not shown). Moreover, no expression of the two analyzed epithelial-mesenchymal transition (EMT) markers, Snail1 and vimentin, was detected in either group. Similarly, the expression and subcellular localization of β-catenin did not change its pattern between groups (data not shown). Additional proteins that are associated with migration, invasion/and or metastases in CRC, such as serpine1, p-P38, p-MAPK, IL8, PTHLH and CD44v6, showed no significant differences in expression in primary tumour between groups (data not shown).

## DISCUSSION

### Development of two CRC models with high metastatic efficiency

We improved the metastatic yield of the HCT116 and SW48 CRC cell lines by using SC passage prior to orthotopic microinjection. In the case of HCT116, this procedure maintained the dissemination pattern at all clinically relevant sites (lymph nodes, liver, lung and peritoneum). In the SW48 cell line, SC preconditioning generated metastases at lymph nodes, lung and peritoneum. This was in contrast to the direct orthotopic microinjection that generated a non-metastatic model. These results are consistent with our previous report of increased metastases in lymph nodes and bone marrow in a diffuse large B-cell lymphoma model after SC conditioning (Bosch et al., 2012), suggesting that this procedure could be used to increase the metastatic yield of the *in-vitro*-established cell lines. In both CRC models, SC preconditioning enhanced, in an organ-specific manner, the number of microfoci (colonization) and/or its growth to macrofoci and visible metastases. In the HCT116 model, this procedure significantly increased the number and area of visible lymph node foci and enhanced colonization of the lung. It also increased colonization in the liver and enhanced the transition from micro- to macro-foci and to visible metastases. Interestingly, the animals with massive liver metastases died earlier than the other animals, a finding that is consistent with compromised liver function and death from liver metastases.

We consider that SC preconditioning prior to orthotopic cell microinjection generates relevant models of metastases because they replicate the situation observed in individuals with CRC, who frequently die of metastases. In the case of the HCT116 model, this is especially relevant because they generate visible metastases that compromise mouse survival, as in humans (Goldberg et al., 1998). SW48 generates metastases at all sites except for the liver, because SW48 cells do not express EGFR, which is necessary for liver colonization (Radinsky et al., 1995; Hewitt et al., 2000). Therefore, after SC preconditioning, both models improve the efficiency of current orthotopic models, which generate a low metastatic rate (Fidler, 1973; Brand et al., 1996; Bruns et al., 1999; Céspedes et al., 2006; Mohanty and Xu, 2010). The fact that metastatic rate is increased in both CRC models suggests that this procedure could be applied to increase metastatic rate in other CRC models and likely in other tumour types.

In addition, this procedure could be useful to study the mechanisms of metastases simultaneously in the several organs and the preclinical development of anti-metastatic drugs. It has the advantage of reducing experimental time as compared with the use of successive *in vivo* passages: our model takes only 15–20 days to develop an SC tumour and about two or three additional months to obtain metastases in the subsequent orthotopic models.

### SC preconditioning increases tumour cell survival and invasion

Metastatic dissemination was probably affected by a process that is common, and prior, to tumour cell dissemination towards all metastatic sites. This argument is in agreement with the higher number of surviving tumour cells (as assessed by the lower number of apoptotic figures observed) in HCT16 SC+ORT and SW48 SC+ORT primary tumours than in their respective ORT groups. It is also consistent with the higher number of keratin-positive single-tumour epithelial cells and higher number of tumour cells forming small clusters (tumour budding) at the invasion front in HCT116 SC+ORT primary tumours. The same trend towards increased invasion was also present in the SW48 SC+ORT tumour edge. However, it did not reach significance. Therefore, whereas in the HCT116 model both increased invasion and reduced tumour cell death can enhance metastatic dissemination, in the SW48 model the enhanced dissemination seems to depend mainly on reduced tumour cell death. In addition, the fact that tumour cell death rate in HCT116 tumours is around fourfold lower than in SW48 is in agreement with the lower metastatic capacity observed in SW48 mice than in HCT116 mice. This lower metastatic capacity of the SW48 model could be explained by the lower viability of these cells, as compared with HCT116 cells, when invading the tissue or disseminating through the bloodstream.

Our findings agree with the enhanced metastatic dissemination and reduced survival associated with detection of single-tumour cells and tumour budding at the tumour invasive front in other CRC mouse models (Gabbert et al., 1985; Shinto et al., 2006) and in CRC patients (Hase et al., 1993; Ueno et al., 2004; Kazama et al., 2006; Prall, 2007; Mitrovic et al., 2012). The decreased apoptosis and increased metastatic rate observed in the SC+ORT as compared to the ORT group, for both CRC models, which were also found when comparing the SW48 model with the HCT116 model, are consistent with the higher metastatic capacity of tumour cells capable of resisting different apoptotic stimuli in their way from the primary tumour to the metastatic sites in cancer models (Townson et al., 2003). They are also in agreement with the prognostic value of apoptotic rate measurement in primary tumours: lower apoptotic index being associated with increased local recurrence, worst local control and shorter overall survival in CRC patients (Adell et al., 2001; Bendardaf et al., 2003; Marijnen et al., 2003; Rupa et al., 2003)

### SC preconditioning alters the expression of proteins that regulate survival and invasion

Detachment of single epithelial cells from the tumour mass has been associated with enhanced migration and invasion, and it affects both lymphatic and haematogenous dissemination (Gabbert et al., 1985; Bacac and Stamenkovic, 2008; Schofield, 2011). We assessed whether SC preconditioning induced changes in the molecular regulation of angiogenesis, cell survival, cell-cell contact and cell adhesion, and whether protease activation leading to ECM degradation could have induced single cell migration. This hypothesis was based on the capacity of cancer cells to modify their migration and invasion mechanisms in response to the environment (Friedl and Wolf, 2003).

VEGF-A was enhanced in the primary tumours of SC+ORT HCT116 mice as compared to ORT HCT116 mice (although these differences were not found in the SW48 model). This is consistent with the overexpression of VEGF mRNA and protein associating with tumour progression and poor prognosis in CRC patients (Hansen and Jakobsen, 2011). It has been reported that the release of VEGF-A and the presence of VEGF receptors in tumour cells establish an autocrine loop and induce a response in stromal cells that favours adhesion, survival, migration and invasion of tumour cells (Calvani et al., 2008; Ahluwalia et al., 2013). It is also known that VEGF-A is a key mediator of angiogenesis (Folkman, 1995). Nevertheless, the differential expression pattern we observed in the HCT116 model between groups was not associated with changes in tumour vasculature (as assayed by CD34, a blood-vessel marker).

The increased number of single tumour epithelial cells observed at the invasive front in SC+ORT primary tumours and its association with higher level of p-AKT might be explained by the previous report that AKT activation increases resistance to apoptosis induced by loss of cell attachment, a function that is required for solitary cell migration and invasion (Grille et al., 2003; Xue and Hemmings, 2013). Moreover, in spite of the increased number of single epithelial cells we observed at the invasive front, the lack of mesenchymal markers in SC+ORT tumours suggests that primary tumours in this group might use a type of single-cell migration not associated with EMT. In addition, in HCT116- and SW48-derived mice, ORT tumours showed low tumour budding and a low number of single tumour cells at the invasive front, together with upregulation of β1 integrin in the case of HCT116. The report that this molecular feature is functionally involved in collective migration in fibrosarcoma (Wolf et al., 2007) or melanoma (Hegerfeldt et al., 2002) cells suggests that tumour cells in our HCT116 model could invade using this migration mechanism, although not necessarily excluding other migration types. Collective migration requires both the establishment of cell-cell contact and the adhesion to the ECM (Yilmaz and Christofori, 2010; Kawauchi, 2012). This is a common migration type in solid tumours (Friedl et al., 2012) and it has also been described in CRC models both *in vitro* and *in vivo* (Nabeshima et al., 1995; Nabeshima et al., 1999; Nabeshima et al., 2000). However, this is a matter of speculation and further studies should be performed to unravel the type of migration used in such models.

In conclusion, to our knowledge, this is the first description of a procedure that increases cell survival and invasion at the tumour invasion front, leading to increased metastatic efficiency at all clinically relevant sites in two CRC models. These models could be useful to simultaneously study the mechanisms of metastases in several organs and might facilitate the preclinical development of novel anti-metastatic drugs.

## MATERIALS AND METHODS

### Cell lines

The HCT116 and SW48 cell lines were purchased from the American Type Culture Collection (ATCC; Manassas, USA) and was cultured in DMEM (ref. 10829018, Invitrogen, UK) supplemented with 10% FBS (ref. F2442, Sigma-Aldrich, St Louis, USA), 50 units/ml penicillin and 50 mg/ml streptomycin (ref. 15140122, Invitrogen).

### SC preconditioning and orthotopic cell microinjection

In HCT116- and SW48-derived tumours, we compared primary tumour growth, invasion and metastasis development between two groups: one group (ORT) received a direct orthotopic microinjection into the cecum of HCT116 and SW48 cells grown *in vitro*, and the other group (SC+ORT) received an orthotopic microinjection of cells disaggregated from SC tumours (HCT116-SC or SW48-SC) previously generated in a different set of mice. We used Swiss nude mice (Charles River, L’Arbresle, France) for *in vivo* studies. The study was approved by the Animal Ethics Committee at Hospital de la Santa Creu i Sant Pau.

We subcutaneously injected 2×10^7^ cells, in DMEM, in both flanks, in five mice. Tumours were measured every 2 days using a caliper. When tumours reached an approximate volume of 700 mm^3^, mice were sacrificed by cervical dislocation and SC tumours were excised. Necrotic areas were discarded. Tumour aliquots were taken for histological study as described (Céspedes et al., 2007), or frozen in liquid nitrogen for molecular studies.

Three hundred mg of viable tumour tissue was then cut into pieces and disaggregated in a mix of 0.05% trypsin (ref. 25300096, Invitrogen) and 100 mg/ml DNase (ref. D5025, Sigma). The mix was pipetted 30 times using a 10 ml pipette, and incubated for 10 minutes at 37°C with shaking. It was then re-pipetted 30 times – using 10 ml, 3 ml and 1 ml pipettes – and re-incubated for 5 minutes at 37°C with shaking. This re-pipetting step was then repeated. The obtained HCT116-SC or SW48-SC single-cell suspension was filtered through a cell strainer and centrifuged at 1000 ***g*** for 10 minutes before counting the cells.

We then randomized 24 female Swiss nude mice, weighing 18–20 g, in two groups: orthotopic (ORT, *n*=8–12) and subcutaneous+orthotopic (SC+ORT, *n*=8–12) mice. We next injected 2×10^6^ HCT116 cells or SW48 cells, previously grown in culture and resuspended in 50 μl of media, in the cecum of each mouse in the ORT group, following the method published by our group (Céspedes et al., 2007). The cecum of each SC+ORT mouse was then injected with 2×10^6^ disaggregated HCT116-SC or SW48-SC cells.

### Analysis of primary tumour growth and metastatic dissemination at necropsy

Mice were monitored every week by palpation and euthanized when they lost 10% of their body weight or showed signs of pain or illness. A complete necropsy was performed, recording the size of the primary tumour, and the number of visually metastatic foci in lymph nodes, liver, lung and peritoneum. Histopathological processing was performed as described previously (Céspedes et al., 2007).

### Histopathological analysis of the primary tumour, and apoptotic and invasive rates at the invasion front

We histologically examined the primary tumours and all organs with expected metastases, to evaluate the degree of differentiation, necrotic areas, apoptotic and mitotic rate, and tumour invasion and vascularization. Three H&E sections for each metastatic site were examined microscopically to identify micro- and macro-metastases in each organ.

We recorded the number and the area of micro- and macroscopic foci found in the affected organs. Foci areas were quantified using CellD Olympus software (v3.3, Olympus, Japan). Metastatic foci were considered macroscopic when their diameter exceeded 1 mm, meaning that the measured area in their tumour sections was higher than 785,000 μm^2^. All foci with a diameter lower than 1 mm were considered microscopic (Folkman, 1983). Visible foci were defined as those reaching a diameter of over 3 mm.

To compare the invasive capacity of the ORT and SC+ORT primary tumours, we counted 40 fields (3–5 sections per tumour) at the tumour invasive front for each tumour group. After staining with anti-A1/A3 keratin, we recorded the number of keratin-positive single cells and keratin-positive tumour cell clusters, containing five or less cells (tumour budding), per 100× tumour field.

H&E primary tumour sections of each group were used to quantify the number of apoptotic cells per field. Ten 400× fields per primary tumour were analyzed.

### Immunohistochemical analysis of primary tumour and metastatic foci

The immunohistochemical analysis (IHC) was done on FFPE tissue. IHC stains were performed on a DAKO Autostainer automated Link48 (DAKO, California, USA) using standard procedures.

The metastatic foci were verified to be human-origin tumour cells (HCT116 or SW48) by using the human-specific antibody anti-MHC-1 (anti-MHC class I; 1:1000; ref. ab134189, Abcam). Samples were incubated with the corresponding primary antibodies using the following dilutions: integrin β1, β2, β3, β4, β5, α1, α2, α3, α4, α5, α6 and αv (1:100; ref. ECM440, Chemicon, Atlanta, USA), p-AKT (1:10; ref. M3628, DAKO), ANGPT2 (1:50; ref. AP10103b, Abgent, San Diego, USA), p-MAPK (1:100; ref. 4676, Cell Signaling, Danvers, MA, USA), p-MAPK-38 (1:100; ref. 9211S, Cell Signaling), vimentin (1:300; ref. M0725, DAKO), PTHLH (1:50; ref. ABIN394303, Abgent), VEGFA (1:1000; ref. ab46154, Abcam, UK), CD44v6 (1:1000; ref. ab78960, Abcam), β-catenin (1:300; ref. M33539, DAKO), serpine1 (1:750; ref. ab28207, Abcam), CXCR4 [1:300, Abcam (clone UMB2; #3108-1)], anti-A1/A3 keratin (1:100; ref. M7003, DAKO), CD34 (ready to use; ref. IR632, DAKO), E-cadherin (1:400; ref. 610182, BD Transduction Laboratories, New Jersey, USA), and then with mouse or rabbit secondary antibodies (EndVision, DAKO). We next incubated the sample with DAB substrate (DAKO) for 5 minutes and contrasted the staining using haematoxylin. IHC evaluation was performed by two independent observers, examining only areas with viable tumour. We analyzed the expression of each antibody in the bulk of the primary tumour, in the invasive front and in the metastatic foci in the ORT and SC+ORT groups. We quantified the percent of stained tumour cells in relation to the total cells and estimated the IHC staining intensity (assigning scores ranging from 1 to 3, with 3 being the maximum intensity). We multiplied both values to obtain a final score of protein expression for each sample. This allowed statistical comparison between groups. Similarly, the number of single cells and cell clusters at the tumour invasive front was quantified by two independent observers.

### Statistical analysis

Mice that did not develop primary tumour or metastases in any organ were excluded from the analysis. The Fisher exact test was used to analyze statistically significant differences between primary tumour and metastatic take rates. The Mann-Whitney test was used to compare tumour size, number of apoptotic figures, single tumour cells, tumour clusters or metastatic foci between groups. Differences in survival between groups were evaluated using Kaplan-Meier curves and the Log-rank test. All quantitative values were expressed as mean ± s.e.m. and the statistical tests were performed using SPSS version 11.0 (IBM, New York, USA). Differences between groups were considered significant at *P*<0.05.

## Supplementary Material

Supplementary Material
