# Supplementary material for: Subcutaneous preconditioning increases invasion and metastatic dissemination in mouse colorectal cancer models
Source: Dis Model Mech. 2014 Jan 30;7(3):387–96. doi: 10.1242/dmm.013995 (PMC3944498; doi:10.1242/dmm.013995)
Supplement: Supplementary Material [file supp_7_3_387__index.html]

Subcutaneous preconditioning increases invasion and metastatic dissemination in mouse colorectal cancer models — Supplementary Material 

# Subcutaneous preconditioning increases invasion and metastatic dissemination in mouse colorectal cancer models

## DMM013995 Supplementary Material

**Files in this Data Supplement:**

- **Supplementary Material**
