# Supplementary material for: Subcutaneous preconditioning increases invasion and metastatic dissemination in mouse colorectal cancer models
Source: Dis Model Mech. 2014 Jan 30;7(3):387–96. doi: 10.1242/dmm.013995 (PMC3944498; doi:10.1242/dmm.013995)
Supplement: Supplementary Material [file supp_7.3.387_DMM013995.pdf]

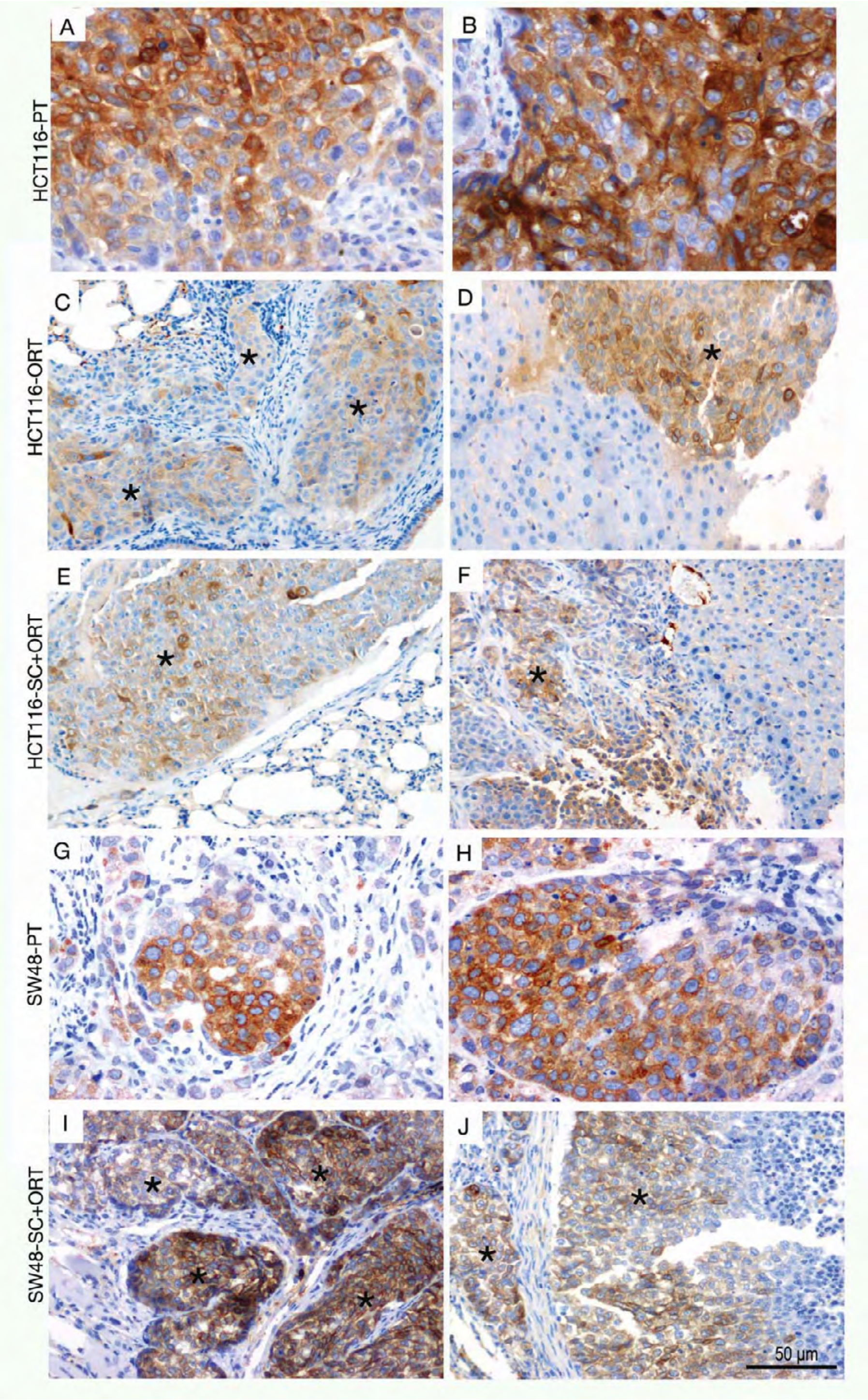

Figure S1. Primary tumours and metastasic foci in HCT116 and SW48 mice express a human specific marker. Primary tumours (A and B) and metastatic foci that developed in lung (C, E) or liver (D, F) in the ORT and SC+ORT group of HCT116 mice expressed anti MHC class I marker, confirming that metastases derived from human CRC cells. Expression of this marker was also observed in primary tumours from SW48 ORT (G) and SW48 SC+ORT (H) and in metastatic foci in diaphragm (E) and mesentery (F) in mice from the SW48 SC+ORT group. Scale bars for magnification (A-H all have the same magnification).

Table S1. Quantitation of VEGFA protein expression in primary tumour observed by Elisa in HCT-116-derived SC+ORT and ORT groups.

**Antibody**	**HCT116 Group**	**pgVEGFA/ug primary tumour protein (mean ±SE)**
VEGFA	ORT	**3.7± 0.7** [a]
	SC+ORT	**14.2± 2.8** [a]

Statistically significant differences observed between groups after applying the Mann-Whitney test: [a] *p=0.004*

Table S2. Quantification of AKT-P and Integrin β1 protein expression in primary tumours observed by IHC in HCT-116 and SW48-derived SC+ORT and ORT groups.

Cell line	Group	Protein expression in primary tumour (mean ±SE)	
		AKT-P	Integrin β1
HCT116	ORT	**17.7± 8.3** [a]	**191.7± 19.8** [b]
	SC+ORT	**70.0± 4.4** [a]	**68.5± 25.1** [b]
SW48	ORT	No expression	No expression
	SC+ORT	No expression	No expression

Abbreviation: ORT, model generated by direct injection of HCT116 and SW48 cells in the cecum; SC+ORT, model generated by injection into the cecum of HCT116-SC and SW48-SC cells obtained by disaggregation of previously generated subcutaneous tumours (SC preconditioning); data expressed as mean ± standard error. Statistically significant differences observed between groups after applying the Mann-Whitney test: [a] *p=0.009*, [b] *p=0.008*
